# Galectin-3 Regulates the Expression of Tumor Glycosaminoglycans and Increases the Metastatic Potential of Breast Cancer

**DOI:** 10.1155/2019/9827147

**Published:** 2019-12-17

**Authors:** Jonathas Xavier Pereira, Sofia Nascimento dos Santos, Thaís Canuto Pereira, Mariana Cabanel, Roger Chammas, Felipe Leite de Oliveira, Emerson Soares Bernardes, Márcia Cury El-Cheikh

**Affiliations:** ^1^Programa de Pós-Graduação em Anatomia Patológica, Faculdade de Medicina da Universidade Federal do Rio de Janeiro, Rio de Janeiro, RJ, Brazil; ^2^Centro de Radiofarmácia, Instituto de Pesquisas Energéticas e Nucleares (IPEN), São Paulo, SP, Brazil; ^3^Instituto de Ciências Biomédicas, Universidade Federal do Rio de Janeiro, Rio de Janeiro, RJ, Brazil; ^4^Programa de Pós-Graduação em Ciências Morfológicas, Instituto de Ciências Biomédicas, Universidade Federal do Rio de Janeiro, Rio de Janeiro, RJ, Brazil; ^5^Laboratório de Oncologia Experimental e Instituto do Câncer do Estado de Paulo, Faculdade de Medicina, São Paulo, São Paulo, SP, Brazil; ^6^Programa de Pós-Graduação em Ciências Aplicadas a Produtos para a Saúde, Faculdade de Farmácia, Universidade Federal Fluminense, Niterói, RJ, Brazil; ^7^Instituto de Ciências Biomédicas, Universidade Federal do Rio de Janeiro, Rio de Janeiro, RJ, Brazil

## Abstract

Galectin-3 (Gal-3) is a multifunctional *β*-galactoside-binding lectin that once synthesized is expressed in the nucleus, cytoplasm, cell surface, and extracellular environment. Gal-3 plays an important role in breast cancer tumors due to its ability to promote interactions between cell-cell and cell-extracellular matrix (ECM) elements, increasing tumor survival and metastatic dissemination. Still, the mechanism by which Gal-3 interferes with tumor cell migration and metastasis formation is complex and not fully understood. Here, we showed that Gal-3 knockdown increased the migration ability of 4T1 murine breast cancer cells *in vitro*. Using the 4T1 orthotopic breast cancer spontaneous metastasis mouse model, we demonstrated that 4T1-derived tumors were significantly larger in the presence of Gal-3 (scramble) in comparison with Gal-3 knockdown 4T1-derived tumors. Nevertheless, Gal-3 knockdown 4T1 cells were outnumbered in the bone marrow in comparison with scramble 4T1 cells. Finally, we reported here a decrease in the content of cell-surface syndecan-1 and an increase in the levels of chondroitin sulfate proteoglycans such as versican in Gal-3 knockdown 4T1 cells both *in vitro* and *in vivo*. Overall, our findings establish that Gal-3 downregulation during breast cancer progression regulates cell-associated and tumor microenvironment glycosaminoglycans (GAGs)/proteoglycans (PG), thus enhancing the metastatic potential of tumor cells.

## 1. Introduction

Breast cancer is the most prevalent cancer among women and has been recognized as a major public health problem worldwide. Metastasis is the leading reason for breast cancer mortality, and several efforts have been made to understand the mechanisms through which tumor cells invade surrounding tissues and distant organs [1]. The interaction of cancer cells with the surrounding tumor microenvironment crucially affects cancer progression. Collagens, laminins, fibronectin, glycosaminoglycans (GAGs), and proteoglycans (PGs) are components of the desmoplastic reaction to tumors and provide a physical barrier against cancer cells [2]. GAGs, in particular, play a key role in tumor progression regulating the adhesion and migration properties of cancer cells, thus modulating their “motile phenotype” [3–7]. GAG molecules like syndecan and versican have been reported to increase the adhesion and invasiveness of primary tumors and have been considered prognostics markers [8, 9].

Galectin-3 (Gal-3) is a *β*-galactoside-binding protein that binds a wide array of glycan-containing glycoproteins expressed on the cell surface and in the extracellular matrix. In the extracellular microenvironment, Gal-3 is able to polymerize and cross-link glycan ligands resulting in the formation of lattice-like structures that have implications in several biological functions [10, 11]. Galectin-3 was reported to interact with chondroitin-4-sulfate and chondroitin-6-sulfate of proteoglycans (CSPGs) [12–14]; however, this interaction was more effective when GAGs were less sulfated. For example, it has been shown that in human prostate cells, Galectin-3 interaction with chondroitin-4-sulfate (C4S) is reduced with the decreased activity of arylsulfatase B (ARSB), an enzyme that removes the 4-sulfate groups from the nonreducing end of chondroitin-4-sulfate (C4S) or dermatan sulfate (DS). Gal-3 was then available to interact with a complex of transcription factors at the AP-1 (activator protein-1) and SP-1 (specificity protein-1) sites on the versican promoter increasing its expression [15]. In another report, Gal-3 silencing in colonic epithelial cells was shown to decrease the transcriptional activity of AP-1 and SP-1 [15–20]. Moreover, a decreased expression of ARSB was associated with an increase in the invasiveness of human melanoma cells by reducing Gal-3 binding to C4S [21]. Thus, the interaction of Gal-3 with glycosylated components of the tumor microenvironment, such as GAGs, may form a physical and functional scaffold having an important role in cancer biology [14, 22]. In fact, the increase in sulfated GAGs during tumor progression can increase the protumoral role of Gal-3 in the nucleus of tumor cells [15].

Our previous studies have shown that primary breast tumors were more aggressive and had a greater potential to metastasize to the bone marrow when injected subcutaneously in Galectin-3 knockout (Lgals3^−/−^) mice [23]. These data suggested that Gal-3 plays a central role, at least in part, in the structural organization of tissues acting as a physical barrier to control cell dissemination. Indeed, a decreased expression of Gal-3 is usually observed during breast cancer progression, while cells embolized in the tumor vasculature present high levels of Gal-3, which indicates that Gal-3 maintains the cohesion between the tumor cells until they find a fertile soil to establish a future metastatic seed [21,24–26]. Although the role of Gal-3 in the maintenance of a tumorigenic phenotype by breast carcinoma cells has been previously demonstrated [27], its role in the tumor biology of breast cancer progression and metastasis is still controversial.

The initial steps of tumor invasion involve tumor cell binding to the extracellular matrix. Since Gal-3 has a broad spectrum of action in cancer biology, both through intracellular and extracellular mechanisms, in this study, we aimed to explore how the differential expression of Gal-3 in tumor cells and its surrounding tumor microenvironment affects tumor invasion and metastasis. We found that a decreased expression of Gal-3 during breast cancer progression increases the metastatic potential of 4T1 murine breast cancer cells to the bone marrow. The increased metastatic potential of silenced Gal-3 breast cancer cells was found to be associated with an overall reduction in the tumor content of GAGs but enhanced chondroitin sulfate A and C, versican, and the matrix metalloproteinase 9 (MMP9) expressions. These data were highlighted when 4T1 cells were grown in Lgal3^−/−^ mice demonstrating that microenvironmental Gal-3 has a further role in controlling GAGs. Our results suggest that Galectin-3 downregulation in tumors during the course of cancer progression, invasion, and further metastasis is intrinsically associated with ECM remodeling, which favors Gal-3-mediated detachment of tumor cells to the primary site via regulation of GAGs.

## 2. Methods

### 2.1. Animals

Female Lgals3^+/+^ and Lgals3^−/−^ Balb/c [28] mice were obtained from the animal facilities of the Nuclear Energy Research Institute (IPEN). All experiments were in compliance with the relevant laws and were approved by the Ethics Committee of Animal Use at the Federal University of Rio de Janeiro (registration number: DAHEICB069) and the Nuclear Energy Research Institute of São Paulo (registration number: 203/17).

### 2.2. Breast Cancer Cell Line

The breast cancer cell line 4T1 was a donation from Dr. Adriana Bonomo (Oswaldo Cruz Institute, FIOCRUZ), Rio de Janeiro, Brazil, and was routinely maintained in RPMI supplemented with 10% of fetal bovine serum. Confluent cell monolayers were subcultured every 3 days and not kept for more than five passages.

### 2.3. Generation and Cloning of Galectin-3 Knockdown 4T1 Cell Clones

Stable shRNA 4T1 cells targeting Gal-3 (TRCN0000029305, Sigma) or the negative control (SHC016, Sigma) were generated after cotransfection of 30 *μ*g of shRNA-containing plasmids with 15 *μ*g pPAX2 and 5 *μ*g of pMDG.2 (Addgene) into HEK293t packaging cell line using the CaCl_2_ method. The viral supernatant was recovered, and the transduced cells were generated by the infection of shRNA lentiviral particles with MOI = 2 (multiplicity of infectious units). On the next day, cells were replaced with fresh medium, and a day later, cells were selected with 1 *μ*g/mL of puromycin for 1 week. After viral transduction 4T1-scramble and 4T1-shRNA-Gal-3 cells were cloned using the limiting dilution method.

### 2.4. *In Vitro* Wound Healing Assay

4T1-scramble or 4T1-shRNA-Gal-3 cells were seeded in 6-well plates to grow in a confluent monolayer. Then, a sterile 20–200 *μ*L pipette tip was held vertically to scratch a line in each well. The detached cells were removed by washing with 1 mL of PBS, and 2 mL of fresh medium was added afterwards and incubated for 48 h. The scratch closure was monitored and imaged after 0 h, 24 h, and 48 h. The cell migration rate (pixel/min) was determined as the slope of the linear regression of the number of cells present in the scratch area over time. The data were analyzed using the GraphPad Prism version 5.0, San Diego, California. Additionally, at zero, 24 and 48 hours after scratching, cells were fixed with methanol and cells were stained with an anti-Ki-67 antibody (Vector), detected with a secondary biotinylated anti-rabbit, ExtrAvidin-Peroxidase (Sigma) and revealed with DAB (DAKO).

### 2.5. Western Blotting

4T1-scramble and 4T1-shRNA-Gal-3 clonal cells were lysed in RIPA buffer, and 50 ug of protein was separated by SDS page (Invitrogen). The content was transferred to a PVDF membrane (Invitrogen), incubated with the primary anti-Gal-3 antibody (M3/38; ATCC, USA), and revealed with the secondary anti-rat antibody conjugated with peroxidase (HRP). Detection was done with chemiluminescence ECL reagent (GE HealthCare), and images were acquired using ImageQuant (GE HealthCare). *β*-Actin was used as a control.

### 2.6. RNA Extraction, Reverse Transcription, and Quantitative PCR

Total RNA from the 4T1-scramble and 4T1-shRNA-Gal-3 cells and from primary tumors was isolated using the Tri-Reagent (Sigma) according to the manufacturer's instructions. Complementary DNA (cDNA) was synthesized from 1 *μ*g of total RNA using the high-capacity cDNA RT kit (Applied Biosystems), according to the manufacturer's protocols. Quantitative PCR analysis was performed in triplicate using Power SYBR Green Master Mix (Applied Biosystems). Relative quantification was done using the ΔΔCt method, normalizing to the *β*-actin gene expression. The primer sequences of mouse *β*-actin, Galectin-3 (Gal-3), syndecan-1 (Sdc1), N-acetyl-galactosaminyltransferase 1 (CSGalNAcT-1), chondroitin polymerizing factor/chondroitin synthase 2 (Chpf), versican, arylsulfatase B (ARSB), and matrix metalloproteinase 9 (MMP9) can be found in Table 1.

### 2.7. Flow Cytometry

1 × 10^6^ 4T1-scramble and 4T1-shRNA-Gal-3 cells were initially incubated with the Fc blocker antibody (clone 2.4G2) for 10 min and then with anti-Gal-3 monoclonal antibody (M3/38; ATCC, USA) for 20 min. After this period, cells were washed with PBS and the secondary anti-rat IgG-FITC (Sigma) was added to cells for 20 min. Cell cycle analysis was performed according to Vindelov's protocol [29]. 4T1-scramble and 4T1-shRNA-Gal-3 cells (1 × 10^6^) were resuspended in 500 *μ*l of propidium iodide solution (PBS, 0.1% Triton X-100, 0.1 % RNAse, 50 *μ*g/ml propidium iodide; Sigma Aldrich, USA). Samples were acquired using the FACScalibur flow cytometer (BD Bioscience, USA) and analyzed in Cell Quest software.

### 2.8. Experimental *In Vivo* Assay

10^5^ 4T1-scramble (SC) or 4T1-shRNA-Gal-3 (SH) cells were inoculated in the fourth mammary fat pad of Balb/c Lgals3^+/+^ (WT) or Lgals3^−/−^ (KO) female mice. The tumor volume was measured three times a week with a pachymeter. In order to calculate accurately the tumor volume, since the mammary tumors seemed to take on an oblate spheroid geometry, we used the following formula: “*V* = (*W*(2) × *L*)/2,” where “*V*” is the tumor volume, “*W*” is the tumor width, and “*L*” is the tumor length, according to Faustino-Rocha et al. [30]. Twenty-eight days after orthotopic injection (p.o.i.), mice were sacrificed, and tumors were excised for mRNA extraction and immunohistochemistry analysis. Each group consisted of five mice, and they were used in each group in two independent experiments.

### 2.9. Detection of Glycosaminoglycans in 4T1-Scramble and 4T1-shRNA-Gal-3 Cells and in 4T1-Derived Tumors

4T1-scramble and 4T1-shRNA-Gal-3 cells were cultured in a 24-glass bottom well plate, and, when confluence was reached, cells were fixed with 4% PFA for 10 min. Twenty-eight days p.o.i., the primary tumors were collected, fixed in 4% PFA for 24 hours, and paraffin-embedded and slices of 5 *μ*m were obtained. Both cells and tissues were stained using 1% Alcian blue 8GX (in 1% of acetic acid) for 30 min according to Wang et al. [31]. The counterstaining was performed using Ponceau 1% in saturated aqueous solution of picric acid and acetic alcohol (1%) for 2 min.

### 2.10. Detection of Syndecan-1 and Chondroitin Sulfate in 4T1-Scramble and 4T1-shRNA-Gal-3 Cells and in 4T1-Derived Primary Tumors

Cells previously grown in a glass bottom well and slices of 5 *μ*m of primary tumors were obtained as described above. For the detection of syndecan-1, samples were incubated with the primary monoclonal antibody (anti-CD138) using a detection “mouse on mouse” Kit (Histofine). For the chondroitin sulfate detection, we used a specific monoclonal antibody (Abcam-ab11570) that reacts specifically with chondroitin-4-sulfate (C4S) and chondroitin-6-sulfate (C6S), but not with the chondroitin-2-sulfate (dermatan sulfate). The photomicrographs were obtained using the software Axioplan®. Staining was quantified by the software TMARKER®, according to Schüffler et al. [32].

### 2.11. Detection of 4T1-Scramble and 4T1-shRNA-Gal-3 Cells in the Iliac Crest Bone Marrow

The iliac crest bone marrow samples were collected from Balb/c female Lgals3^+/+^ and Lgals3^−/−^ previously inoculated with 4T1-scramble and 4T1-shRNA-Gal-3 cells, as described previously [23] and were fixed in 4% PFA for 24 hours. After this period, samples were decalcified in EDTA 20% during 14 days and embedded in paraffin. The double staining performed in the iliac crest bone marrow samples for proliferative nuclear cell antigen (anti-PCNA, DAKO) and cytokeratin-19 (anti-CK-19, Abcam) was performed using a standard immunohistochemistry protocol, where proliferative nuclei were visualized with 3,3′-diaminobenzidine (DAB; Spring Bioscience) staining while cytokeratin-19 was visualized with nitro-blue tetrazolium chloride (NBT) and 5-bromo-4-chloro-3′-indolyphosphate p-toluidine salt (BCIP), according to Silva dos Santos et al. [33]. Three animals per group were used in two independent experiments. Quantification of positive cells per section was done in 5 different fields.

### 2.12. Statistical Analysis

The statistical tests were accomplished using Tukey's multiple comparison test (*t*-test), and significance threshold was fixed for *α* = 0.05. *P* values ≤0.05 were considered statistically significant.

## 3. Results

### 3.1. Galectin-3 Downregulation Modified 4T1 Cell Morphology

**Figure 1:**
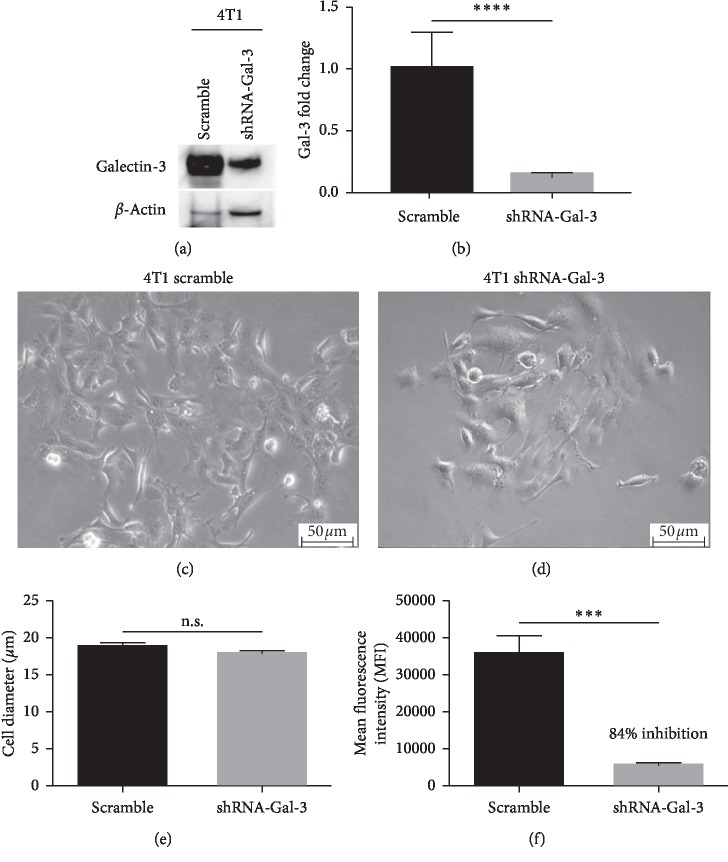
Galectin-3 knockdown modifies the cellular morphology of 4T1 cells. (a) Western blot for Gal-3 in 4T1-scramble cells and 4T1-shRNA-Gal-3 4T1 cells. *β*-Actin was used as loading control. (b) Quantitative PCR for Gal-3 in 4T1-scramble and 4T1-shRNA-Gal-3 cells. Morphology of 4T1-scramble cells (c) and 4T1-shRNA-Gal-3 cells (d) by phase contrast microscopy. (e) Cell diameter of 4T1-scramble cells and 4T1-shRNA-Gal-3 cells obtained by morphometry. (f) Relative amount of Galectin-3 by flow cytometry in 4T1-scramble cells and 4T1-shRNA-Gal-3 cells. Results are shown as means ± s.d. ^*∗∗∗*^*P* < 0.001; ^*∗∗∗∗*^*P* < 0.0001; n.s. means no statistical significance. Magnification: (c, d) 200x. Results of three independent experiments were performed with *n* = 5 for each.

To evaluate the role of Gal-3 in 4T1 cell lines, we initially knocked down Gal-3 in 4T1 cells using a stable shRNA for Gal-3, and then, by clonal selection (data not shown), we isolated the clone with the more prominent inhibition of Gal-3 for further studies (Figures 1(a) and 1(b)). A morphological analysis showed that *in vitro*, the progeny of 4T1-scramble cells was more spread and dispersed in the plastic dish (Figure 1(c)) compared to 4T1-shRNA-Gal-3 cells that were rounder and less dispersed (Figure 1(d)).

These observations suggest that Gal-3 knockdown modified at least in part the cell contact with the substrate. Although rounder and less dispersed, the 4T1-shRNA-Gal-3 cells presented the same diameter compared with 4T1-scramble ones (Figure 1(e)). It is worth to mention that the cell cycle was not altered in both cells (Supplementary [Supplementary-material supplementary-material-1]). The cell-surface expression of Gal-3, evaluated by a flow cytometry analysis, decreased by 85% in 4T1-shRNA-Gal-3 cells compared with 4T1-scramble cells (Figure 1(f)).

### 3.2. Galectin-3 Downregulation Increased 4T1 Cell Invasion *In Vitro*

**Figure 2:**
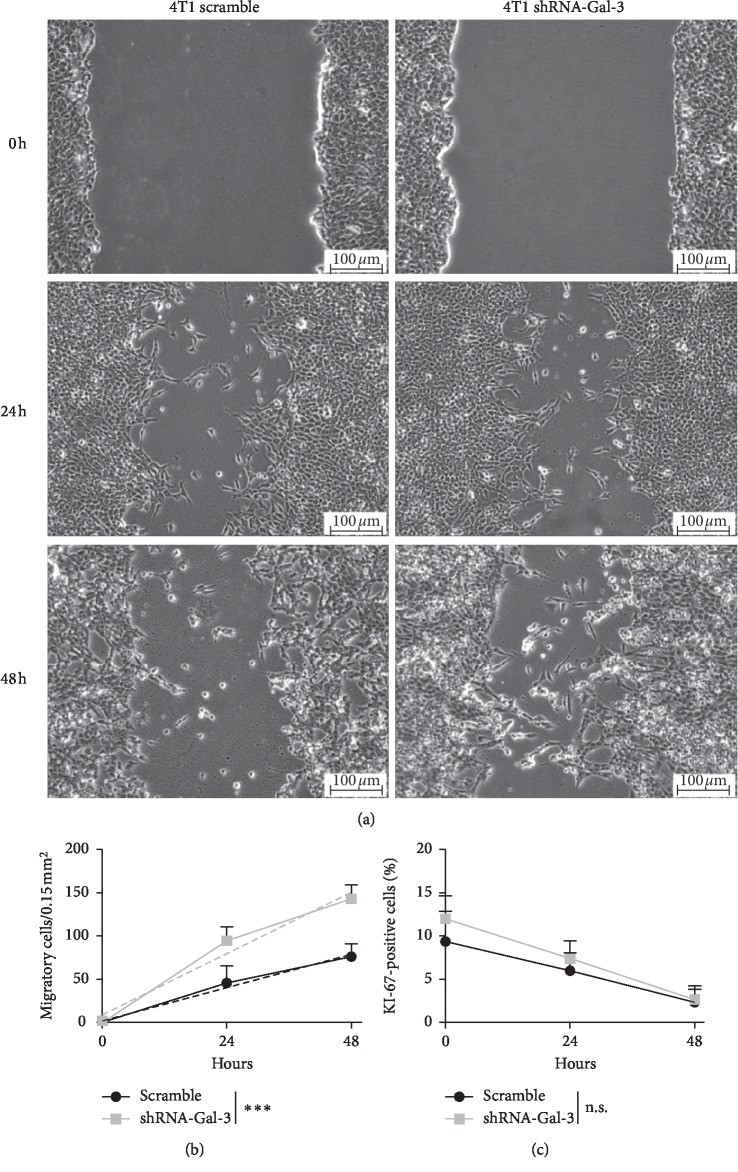
Galectin-3 downregulation increases migration of 4T1 cells *in vitro*. (a) Scratch assay in 4T1-scramble cell culture and 4T1-shRNA-Gal-3 cell culture at zero, 24, and 48 hours. (b) Rate of migration estimated by a linear regression obtained by the number of 4T1-scramble or 4T1-shRNA-Gal-3 cells present on the scratch area. (c) Rate of proliferation estimated by immunostaining for Ki-67 over time in the scratch assay. Results are shown as means ± s.d. ^*∗∗∗*^*P* < 0.001; n.s. means no statistical significance. Magnification: (a) 40x. Results of three independent experiments were performed with *n* = 5 for each.

We next investigated the ability of Gal-3 scramble and knockdown 4T1 cells to migrate in a scratch assay performed in a confluent cell culture. We observed a statistically significant increased ability of 4T1-shRNA-Gal-3 cells to migrate after 24 and 48 hours in comparison with 4T1-scramble cells (Figures 2(a) and 2(b)). To evaluate whether this difference was due to an increase in the proliferation rate of 4T1 knockdown cells, at the end of the migration experiment, cells were stained with Ki-67 (Supplementary [Supplementary-material supplementary-material-1]). No difference in the proliferative rate was found between both cells (Figure 2(c)). Altogether, these data indicate that Gal-3 silencing increases the migration of 4T1 breast cancer cells but not its proliferative capacity.

### 3.3. Galectin-3 Downregulation Reduced Breast Cancer Growth Rate and Increased Bone Marrow Metastasis

**Figure 3:**
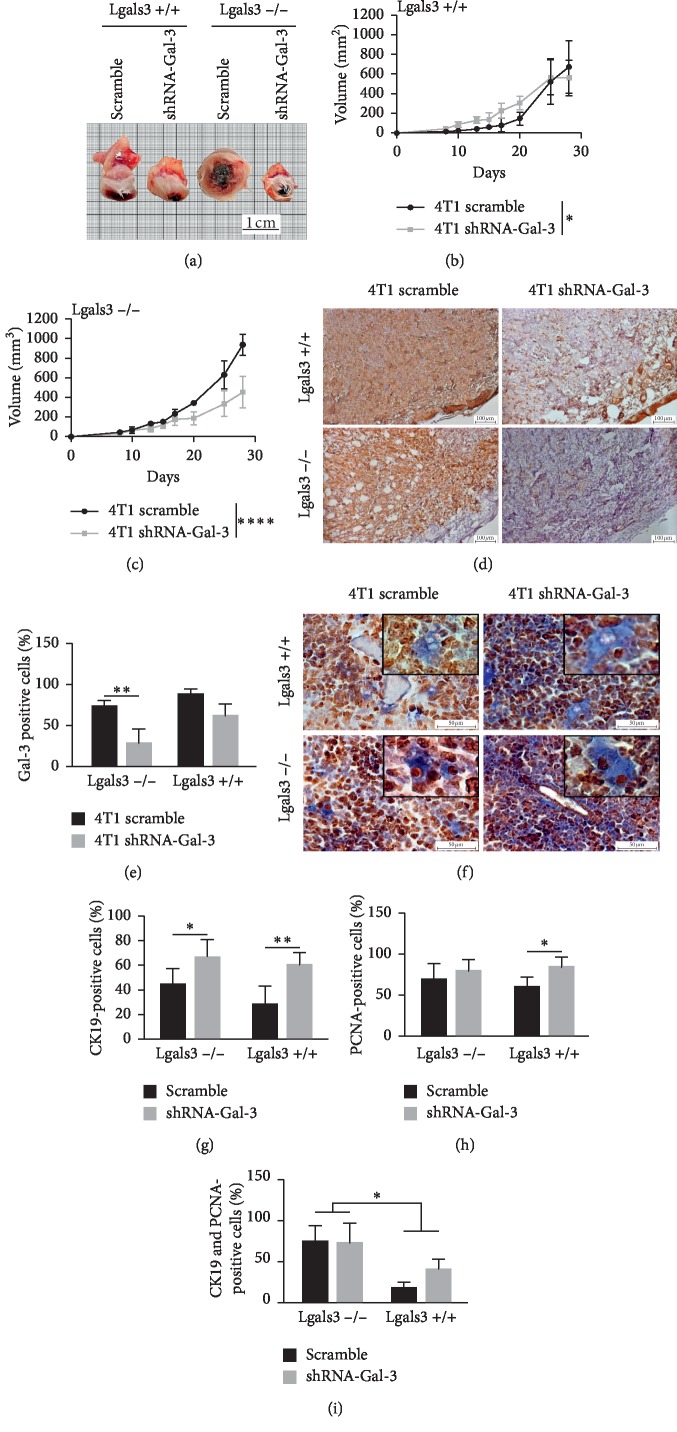
Interplay between tumor and microenvironmental Galectin-3 regulates breast cancer tumor growth and metastasis. (a) Macroscopy of tumors developed by 4T1-scramble cells and 4T1-shRNA-Gal-3 cells in Lgals3^+/+^ and Lgals3^−/−^ mice. Morphometric analysis of tumors developed by 4T1-scramble cells and 4T1-shRNA-Gal-3 cells over 28 days in Lgals3^+/+^ (b) and Lgals3^−/−^ (c) mice. (d) Immunostaining for Galectin-3 in primary tumors developed by 4T1-scramble cells and shRNA-Gal-3 4T1 cells in Lgals3^+/+^ and Lgals3^−/−^ mice. (e) Quantification of Galectin-3 immunostaining in primary tumors. (f) Double staining for cytokeratin-19 (cytoplasm in blue) and PCNA (nuclei in brown) in the bone marrow of Lgals3^+/+^ and Lgals3^−/−^ mice with tumors developed by 4T1-scramble cells and shRNA-Gal-3 4T1 cells. (g) Quantification of total cytokeratin-19 immunostaining in the bone marrow of Lgals3^+/+^ and Lgals3^−/−^ mice with tumors developed by 4T1-scramble cells and 4T1-shRNA-Gal-3 cells. (h) Quantification of total PCNA immunostaining in the bone marrow of Lgals3^+/+^ and Lgals3^−/−^ mice with tumors developed by 4T1-scramble cells and 4T1-shRNA-Gal-3 cells. (i) Double positive staining for cytokeratin-19 and PCNA in the bone marrow of Lgals3^+/+^ and Lgals3^−/−^ mice with tumors developed by 4T1-scramble cells and 4T1-shRNA-Gal-3 cells. Data are representative of two independent experiments using 3–5 animals per experiment (d, e). Data are representative of two independent experiments using 3 animals per experiment (f, h). Five fields per section were quantified. Results are shown as means ± s.d. ^*∗*^*P* < 0.05; ^*∗∗*^*P* < 0.005; ^*∗∗∗∗*^*P* < 0.0001.

We then evaluated the relevance of tumor and microenvironmental Gal-3 to breast cancer biology and performed an orthotopic injection of 4T1-scramble (SC) or 4T1-shRNA-Gal-3 (SH) cells in Lgals3^+/+^ (WT) or Lgals3^−/−^ Balb/c female mice. Tumors derived from 4T1-shRNA-Gal-3 cells were smaller than 4T1-scramble-derived tumors 28 days after orthotopic injection (p.o.i.) regardless of the mice background (Figure 3(a)). When 4T1-shRNA-Gal-3 cells were grown in Lgals3^+/+^ mice (Figure 3(b)), the growth rate was reduced in comparison with 4T1-scramble cells. This difference was more significant when 4T1 cells were injected in Lgals3^−/−^ mice, where we could observe a marked decrease in the tumor growth of 4T1-shRNA-Gal-3 in comparison with 4T1-scramble cells (Figure 3(c)). At the end of the experiment, tumors were collected, and Gal-3 expression was analyzed by immunohistochemistry (Figures 3(d) and 3(e)). We could observe a reduction in Gal-3 expression in 4T1-shRNA-Gal-3-derived tumors in comparison with 4T1-scramble tumors, supporting the stability of the clone generated after silencing. We next assessed whether the interplay between microenvironmental and tumor Gal-3 would influence bone marrow metastasis. Therefore, we performed the double staining for CK-19 (cytoplasm in blue) and PCNA, a marker of cell proliferation (nuclei in brown), in the bone marrow of mice 28 days p.o.i (Figure 3(f)). We found a statistically significant increased number of 4T1-shRNA-Gal-3 cells in both Lgals3^+/+^ and Lgals3^−/−^ bone marrow microenvironments (Figure 3(g)) compared with 4T1-scramble cells. However, the percentage of PCNA-positive cells were only increased when 4T1-shRNA-Gal-3 cells were grown in Lgals3^+/+^ mice microenvironment (Figure 3(h)). Finally, double positive tumor cells (CK-19^+^ and PCNA^+^) were present in greater numbers in the bone marrow of Lgals3^−/−^ mice, regardless of the expression of Gal-3 in 4T1 cells (Figure 3(i)).

Altogether, these data indicate that a reduction in the expression of Gal-3 in tumor cells during breast cancer progression might favor a more metastatic phenotype.

### 3.4. Galectin-3 Downregulation Decreased the Overall Expression of GAGs in Breast Cancer

**Figure 4:**
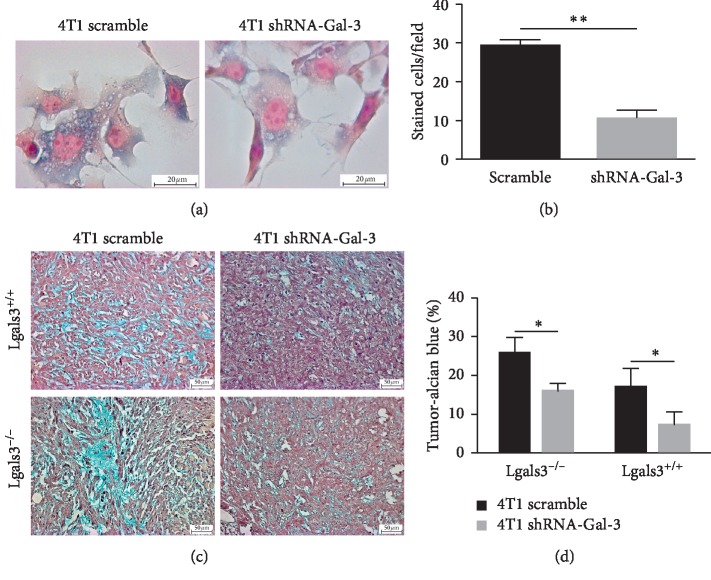
Downregulation of Galectin-3 in breast cancer cells decreases the expression of GAGs both *in vitro* and *in vivo*. (a) Cytochemistry for glycosaminoglycans (Alcian blue) in 4T1-scramble cells and 4T1-shRNA-Gal-3 cells. (b) Quantification of glycosaminoglycans by cytochemical analysis. (c) Histochemistry for glycosaminoglycans (Alcian blue) in primary tumors developed by 4T1-scramble cells and 4T1-shRNA-Gal-3 cells in Lgals3^+/+^ and Lgals3^−/−^ mice. (d) Quantification of glycosaminoglycans by histochemical analysis. Data are representative of two independent experiments using 3–5 animals per experiment (c, d). Results are shown as means ± s.d. ^*∗*^*P* < 0.05; ^*∗∗*^*P* < 0.005.

We then investigated how a reduction in Gal-3 expression triggers tumor cells to metastasize to secondary organs and investigated the overall GAGs content in 4T1 cells and derived tumors. As observed in Figure 4(a), the number of cells stained with Alcian blue (GAGs) was decreased in 4T1-shRNA-Gal-3 cells compared to 4T1-scramble cells (Figure 4(b)). Likewise, the percentage of Alcian blue positive staining in tumors was significantly lower in 4T1-shRNA-Gal-3-derived tumors regardless of the mice background (Figures 4(c) and 4(d)). Therefore, downregulating Gal-3 decreased the total content of GAGs during tumor progression.

### 3.5. Galectin-3 Downregulation Increased the Synthesis of Chondroitin Sulfate

**Figure 5:**
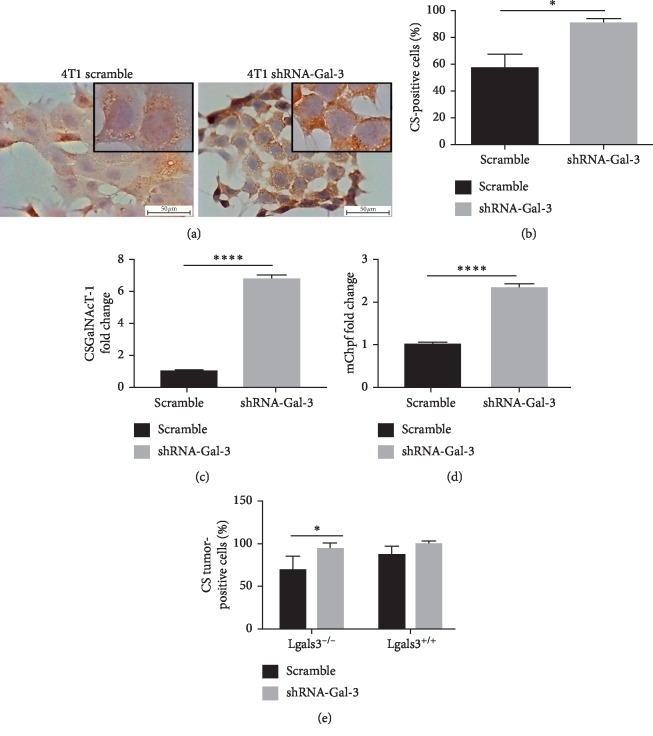
Galectin-3 knockdown increases the synthesis of chondroitin sulfate A and C. (a) Immunostaining for chondroitin-4-sulfate and chondroitin-6-sulfate in 4T1-scramble cells and 4T1-shRNA-Gal-3 cells. (b) Quantification of chondroitin-4-sulfate and chondroitin-6-sulfate immunostaining in 4T1-scramble cells and 4T1-shRNA-Gal-3 cells. (c) Quantitative PCR for N-acetyl-galactosaminyltransferase 1 (CSGalNAcT-1) in 4T1-scramble cells and 4T1-shRNA-Gal-3 cells. (d) Quantitative PCR for chondroitin polymerizing factor/chondroitin synthase 2 (Chpf) in 4T1-scramble cells and 4T1-shRNA-Gal-3 cells. (e) Quantification of chondroitin-4-sulfate and chondroitin-6-sulfate immunostaining in primary tumors developed by 4T1-scramble cells and 4T1-shRNA-Gal-3 cells in Lgals3^+/+^ and Lgals3^−/−^ mice. Data are representative of two independent experiments using 3–5 animals per experiment (e). Results are shown as means ± s.d. ^*∗*^*P* < 0.05; ^*∗∗∗∗*^*P* < 0.0001.

Because of the deregulated balance of GAGs in 4T1-shRNA-Gal-3 cells and derived tumors, we then investigated the content of C4S and C6S in 4T1 cells and derived tumors by immunohistochemistry. Gal-3 knockdown cells presented an increased expression of C4S and C6S in comparison with 4T1-scramble cells (Figures 5(a) and 5(b)). The increase of C4S and C6S in 4T1-shRNA-Gal-3 cells was accompanied by an upregulation of the mRNA levels of N-acetyl-galactosaminyltransferase 1 (CSGalNAcT-1) (Figures 5(c)) and chondroitin polymerizing factor/chondroitin synthase 2 (Chpf) (Figure 5(d)). These enzymes are involved in the synthesis and elongation of CS, respectively, and might be associated with increased CS levels. Interestingly, the total content of C4S and C6S was only significantly higher in 4T1-shRNA-Gal-3-derived tumors in comparison with 4T1-scramble cells grown in Lgals3^−/−^ background mice (Figure 5(e)), whereas no difference was observed in Lgals3^+/+^ mice. Thus, a downregulation of Gal-3 might positively impact the expression of CS.

### 3.6. Galectin-3 Downregulation Decreased the Synthesis of Syndecan-1

**Figure 6:**
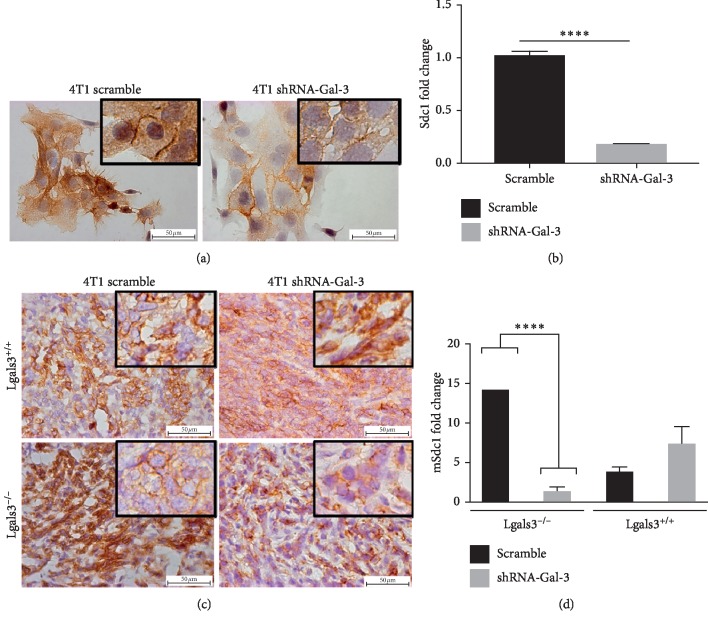
Galectin-3 knockdown decreases syndecan-1 expression. (a) Immunostaining for syndecan-1 in 4T1-scramble cells and 4T1-shRNA-Gal-3 cells. (b) Quantitative PCR for syndecan-1 in 4T1-scramble cells and 4T1-shRNA-Gal-3 cells. (c) Immunostaining for syndecan-1 in primary tumors developed by 4T1-scramble cells and 4T1-shRNA-Gal-3 cells in Lgals3^+/+^ and Lgals3^−/−^ mice. (d) Quantitative PCR for syndecan-1 in primary tumors developed by 4T1-scramble cells and 4T1-shRNA-Gal-3 cells in Lgals3^+/+^ and Lgals3^−/−^ mice. Data are representative of two independent experiments using 3–5 animals per experiment (c, d). Results are shown as means ± s.d. ^*∗∗∗∗*^*P* < 0.0001.

Syndecan-1 (Sdc1) is a cell-surface proteoglycan predominantly involved in cell adhesion and migration of cells. Therefore, we next investigated whether or not the expression of Sdc1 was regulated by Gal-3. 4T1-scramble cells presented higher protein and mRNA levels of Sdc1 in comparison with 4T1-shRNA-Gal-3 (Figures 6(a) and 6(b)). 4T1-derived tumors showed that the protein and mRNA levels of Sdc1 were increased in 4T1-scramble-derived tumors in comparison with 4T1-shRNA-Gal-3 when grown in Lgals3^−/−^ background mice (Figures 6(c) and 6(d)). On the other hand, we could observe a slight increase in Sdc1 levels in 4T1-shRNA-Gal-3-derived tumors compared to 4T1-scramble-derived tumors in Lgals3^+/+^ mice. Altogether, these results suggest that downregulation of Gal-3 in 4T1 cells modifies the behavior of tumor in terms of adhesion between tumor cells and ECM components.

Galectin-3 downregulation during breast cancer progression regulated tumor and microenvironmental GAGs and contributed to the metastatic phenotype.

**Figure 7:**
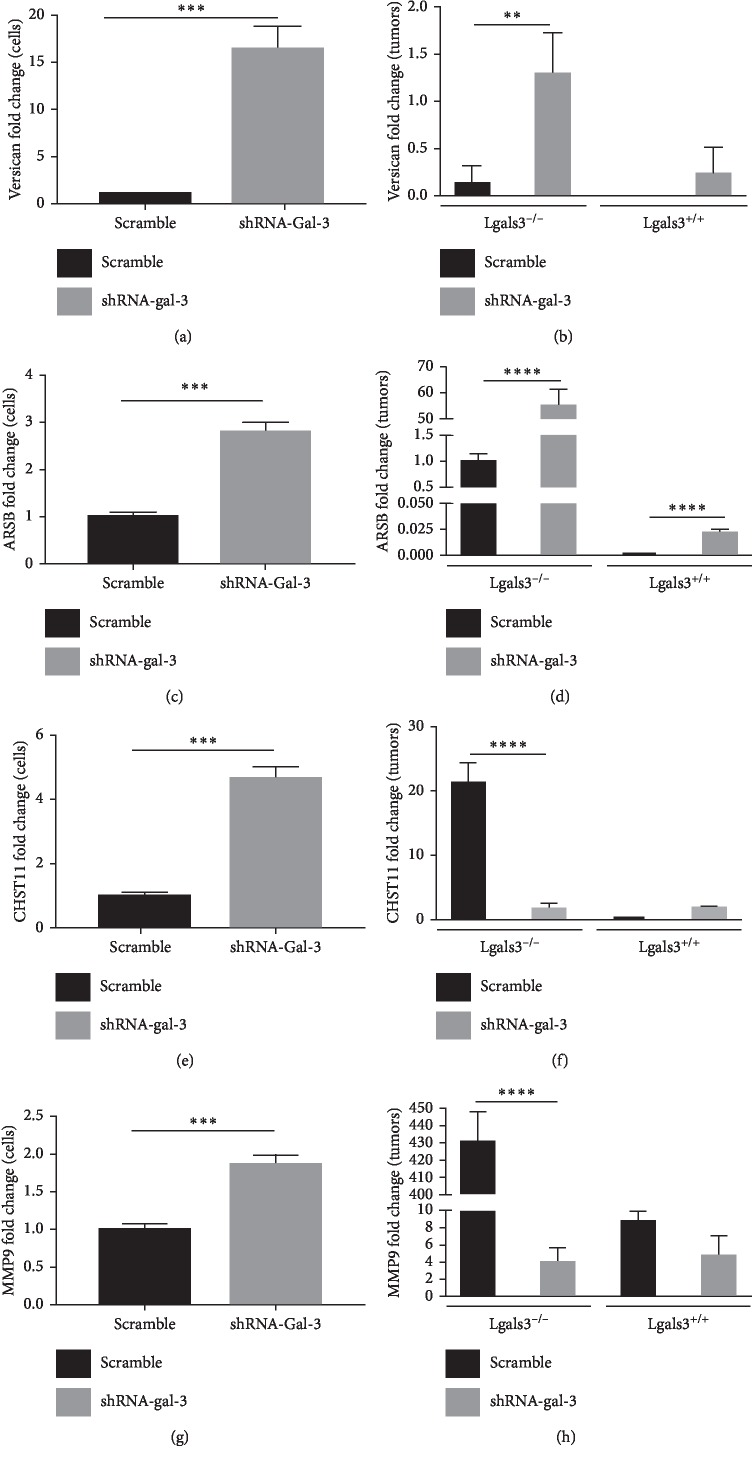
Galectin-3 regulates the mRNA levels of versican, sulfatase, sulfotransferase, and the matrix metalloproteinase 9. (a) Quantitative PCR for versican in 4T1-scramble cells and 4T1-shRNA-Gal-3 cells. (b) Quantitative PCR for versican in primary tumors developed by 4T1-scramble cells and 4T1-shRNA-Gal-3 cells in Lgals3^+/+^ and Lgals3^−/−^ mice. (c) Quantitative PCR for arylsulfatase B (ARSB) in 4T1-scramble cells and 4T1-shRNA-Gal-3 cells. (d) Quantitative PCR for arylsulfatase B (ARSB) in primary tumors developed by 4T1-scramble cells and 4T1-shRNA-Gal-3 cells in Lgals3^+/+^ and Lgals3^−/−^ mice. (e) Quantitative PCR for carbohydrate sulfotransferase 11 (CHST11) in 4T1-scramble cells and 4T1-shRNA-Gal-3 cells. (f) Quantitative PCR for carbohydrate sulfotransferase 11 (CHST11) in primary tumors developed by 4T1-scramble cells and 4T1-shRNA-Gal-3 cells in Lgals3^+/+^ and Lgals3^−/−^ mice. (g) Quantitative PCR for metalloproteinase 9 (MMP9) in 4T1-scramble cells and 4T1-shRNA-Gal-3 cells. (h) Quantitative PCR for matrix metalloproteinase 9 (MMP9) in primary tumors developed by 4T1-scramble cells and 4T1-shRNA-Gal-3 cells in Lgals3^+/+^ and Lgals3^−/−^ mice. Data are representative of two independent experiments using 3–5 animals per experiment (b, d, f, and h). Results are shown as means ± s.d. ^*∗∗*^*P* < 0.005; ^*∗∗∗*^*P* < 0.001; ^*∗∗∗∗*^*P* < 0.0001.

To further explore the molecular mechanisms behind Gal-3 regulation of GAGs, we then investigated whether versican, an important PG involved in cell motility and invasion, was regulated by Gal-3. As observed in Figure 7(a), the mRNA levels of versican were upregulated in 4T1-shRNA-Gal-3 cells compared to 4T1-scramble cells. Likewise, in the absence of microenvironmental Gal-3 (Lgals3^−/−^ mice), 4T1-shRNA-Gal-3-derived tumors presented higher mRNA levels of versican in comparison with 4T1-scramble-derived tumors while no difference in versican mRNA levels was found in Lgals3^+/+^ mice (Figure 7(b)).

Because Gal-3 has a high affinity to less sulfated GAGs, we also investigated the expression of arylsulfatase B (ARSB) and carbohydrate sulfotransferase 11 (CHST11), which are enzymes involved in removal and addition of sulfate groups, respectively, from CS and dermatan sulfate. As seen in Figures 7(c) and 7(d), the mRNA levels of ARSB were upregulated in 4T1-shRNA-Gal-3 cells (Figure 7(c)) and 4T1-shRNA-Gal-3-derived tumors (Figure 7(d)) in comparison with 4T1-scramble cells and tumors. Still, the overall mRNA levels of ARSB were higher when tumors were grown in Lgals3^−/−^ mice compared to Lgals3^+/+^. Also, the mRNA levels of CHST11 were upregulated in 4T1-shRNA-Gal-3 cells when compared to 4T1-scramble cells (Figure 7(d)). Interestingly, in the absence of Gal-3 in the tumor microenvironment (Lgals3^−/−^), there was a reduction in the mRNA levels of CHST11 in 4T1-shRNA-Gal-3-derived tumors in comparison with 4T1-scramble-derived tumors (Figure 7(f)).

Finally, the expression levels of the matrix metalloproteinase 9 (MMP9) were also evaluated since its expression is highly associated with a metastatic phenotype in breast cancer. MMP9 mRNA levels were increased in 4T1-shRNA-Gal-3 cells compared to 4T1-scramble ones (Figure 7(g)), while drastically decreased in 4T1-shRNA-Gal-3-derived tumors developed in Lgals3^−/−^ mice in comparison with 4T1-scramble-derived tumors (Figure 7(h)). Altogether, these data suggest that a decreased expression of Gal-3 during breast cancer progression might render tumor cells less adherent and more migratory by regulating the expression of tumor GAGs and MMP9, thus increasing the metastatic potential of the tumor.

## 4. Discussion

Galectin-3 is one of the most studied galectins, and numerous reports have demonstrated that Gal-3 is directly associated with oncogenesis, angiogenesis, cancer progression, and metastasis. The molecular mechanisms by which Gal-3 regulates tumor invasion and metastasis are greatly influenced by the tumor niche. Gal-3 is known to influence cell-cell interactions, receptor activity on the cell surface, and cell-extracellular matrix interactions, through binding partners such as MUC1, CD44, selectins, or integrins, which are characteristics of metastatic cells.

**Figure 8:**
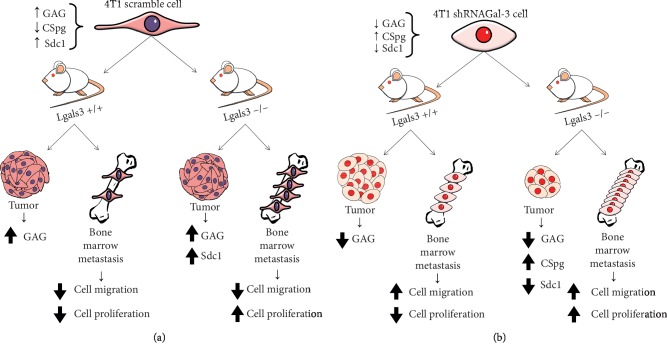
Schematic illustration of the overall findings. (a) The absence of Gal-3 in the microenvironment increased the number of the tumor cells but not its ability to migrate to the bone marrow. (b) In contrast, the low level of intracellular Gal-3 in tumor cells modified not only the migratory capacity of cells but also the number of metastatic cells in a bone marrow.

Here, we showed that Gal-3 downregulation over the course of breast cancer might modify the morphology, adhesiveness, and migratory ability of cancer cells while remodeling the content of extracellular glycosaminoglycans, but not its proliferative capacity. These changes were drastically reflected in the tumor cell biology and reduced the growth rate of 4T1-derived tumor while increasing metastasis to the bone marrow (Figure 8 summarizes our findings).

So far, there is no particular mechanism explaining how Gal-3 regulates the majority of its cellular functions. Galectin-3′s ability to form cell-surface lattices is known to regulate signaling threshold [34], determine the residency time and duration of signaling of cell-surface glycoproteins [35], and activation of downstream signaling pathways [36]. Therefore, disrupting the Gal-3-generated lattice by changes in the glycosylation status of cells accounts for many actions of Gal-3 such as the increased resistance of cancer cells to chemotherapeutics [37] or survival of T cells [38]. We therefore can hypothesize that a decreased expression of Gal-3 over the course of breast cancer progression might be influencing the residency time of cell-surface receptors, which may indirectly lead to a deregulation in the expression levels of enzymes involved in the synthesis of GAGs of CSPG.

Galectin-3 is a *β*-galactoside-binding protein that specifically interacts with LacNAc or poly-LacNAc chains of N- and O-linked oligosaccharides [39]. This lectin has been reported to cross-link, preferentially and with a higher affinity, the nonsulfated regions of GalNAc containing GAGs such as chondroitin-4-sulfate and chondroitin-6-sulfate of proteoglycans (CSPGs) in carbohydrate-dependent manner [15]. Moreover, it was found that GAG binding was not a common characteristic of all galectins since Galectin-1 does not interact with GAGs [13]. Since sulfated GAGs are able to bind bioactive molecules involved in cell-cell and cell-ECM interactions [40], we could assume that the interplay and binding between Gal-3 and GalNAc containing GAGs might be the molecular basis that regulates several biological functions involved with cancer cell migration and invasion.

There is a wide body of evidence suggesting a protumoral role of CS in the migration, proliferation, and metastasis of cancer cells [41–44]. Accordingly, 4T1-shRNA-Gal-3 cells and derived tumors presented increased cellular migration and metastatic cells in the bone marrow, respectively. Considering a wild-type situation, which can mimic the tumor microenvironment of human patients, several reports have shown a direct link between the expression of CHST11 (involved in decorating CSPG4 with chondroitin-4-sulfate) and the increased metastatic potential of breast cancer cells [42, 45]. Also, it is well known that during breast cancer progression, Gal-3 is often downregulated in the primary tumor, contributing to invasion of surrounding areas and migration to blood vessels [26]. Therefore, in a WT condition where the levels of Gal-3 are gradually decreasing in tumor cells, the upregulation of CHST11 might contribute to the overall metastatic behavior of cancer cells. Indeed, the physiological significance of sulfated GAGs is still not fully understood. The patterns of sulfation at different stages of cancer are difficult to predict, but it is clear that modified GAGs affect tumor progression, invasion, and metastasis. Still, Kaiathas et al. [46] reported that chondroitin-4-sulfotransferase-I (the enzyme responsible for the sulfation of C4S) appeared to be decreased with increased stages of colorectal cancer. On the other hand, the same report showed that Chpf (chondroitin synthases) was highly expressed in the tumor specimens compared to healthy tissue. Since sulfation of CS has been reported to decrease Gal-3 affinity for CS [17, 47], we can hypothesize that a reduction in Gal-3 levels in the primary tumor over the course of tumor progression, concomitantly with an increase in CHST11 and decrease in ARSB expression, might contribute to a delocalization of Gal-3 to the intracellular milieu of tumor cells. This could increase cellular motility and metastasis while protecting cancer cells from proapoptotic events and anoikis (a function of intracellular Galectin-3) [48].

It has been reported that the expression of Sdc1 is lower in highly metastatic cells and leads to an increased activation of *β*1-integrins and focal adhesion kinase, which contributes to breast cancer cell adhesion and invasion from the primary tumor [8, 49]. Similarly, we could observe that tumor cells more prone to metastasize, such as 4T1-shRNA-Gal-3 cells, presented decreased levels of Sdc1. Moreover, during the metastatic process, inside blood vessels, breast cancer cells have been reported to reexpress cytoplasmic Galectin-3, contributing to their attachment to blood vessels [26]. Alongside, 4T1 cells were shown to express the vascular receptor P‐selectin ligands on the cell surface and that sulfated CS GAG chains were involved in P‐selectin-mediated adhesion of 4T1 cells to the endothelium [50]. These data suggest an interplay between Gal-3 and the CS sulfation status of tumor cells that may contribute to a superior metastatic potential.

Gal-3 is present in very low quantities after its silencing and interacts highly with poorly sulfated chondroitin in the cytoplasm, as proposed by Bhattacharyya et al. [18, 19]. Accordingly, our results showed that increased arylsulfatase B (ARSB) was accompanied by a decrease in metalloproteinases in tumors, indicating that Gal-3 could be associated with less sulfated GAGs in the cytoplasm and more in the nucleus [18].

Our data indicated that breast tumors developed by Gal-3 knockdown 4T1 cells were more metastatic due to the decrease of cell adhesiveness, facilitating its migration, independent of the metalloproteinase 9 degradation. In our previous studies, we show that the absence of Gal-3 in the microenvironment favored the primary tumor growth and metastasis, demonstrating the importance of extracellular Gal-3 in the tumor biology [23]. In this study, we presented for the first time that the absence of microenvironmental Gal-3 modulated negatively the carbohydrate sulfotransferase 11 (CHST11) mRNA levels in the primary tumors, indicating that the tumor cell line behavior can be modified by the tumor microenvironment.

In conclusion, Gal-3 may have an important role in regulating the availability of GAGs involved in invasion and metastasis, and therefore, decreased sulfation of GAGs could be a marker of a poor prognosis in breast cancer.

## Figures and Tables

**Table 1:** Primer sequences.

Primer	Forward 5′—3′	Reverse 5′—3′
*β*-Actin	CTAAGGCCAACCGTGAAAAG	ACCAGAGGCATACAGGGACA
Gal-3	GGTGGAGCACTAATCAGGAAA	CGGATATCCTTGAGGGTTTG
Sdc1	GAGGGCTCTGGAGAACAAGA	TGTGGCTCCTTCGTCCAC
CSGalNAcT-1	GCGTAATCTACGGCCATCA	TCCTGTTTCCTTCTTTATGACCA
Chpf	CTCGTGTCTTGCCCTACCAT	CGTGCTGATATACCGAGTTCTG
Versican	TCCTGATTGGCATTAGTGAAGA	TTTGTTTTGCAGAGATCAGGTC
ARSB	CAAAAATTGGAAACTCCTCACG	CTCAGAGACGTTGGACTGAGAC
CHST11	CGGAAGGGATCGAGAAGTC	GATGGCAGTGTTGGATAGCTC
MMP9	AGACGACATAGACGGCATCC	TCGGCTGTGGTTCAGTTGT

## Data Availability

The data used to support the findings of this study are included within the article.

## Supplementary Materials

Supplementary Materials Supplementary Figure 1: Galectin-3 knockdown 4T1 cells did not alter the cell cycle. (A) Cell cycle analysis by flow cytometry using propidium iodide (PI) in 4T1-scramble cells and 4T1-shRNA-Gal-3 cells. Results are shown as means ± s.d. Supplementary Figure 2: cell proliferation over time in the scratch assay. Immunostaining for Ki-67 over time in the scratch assay. The red arrows indicate nuclei positive for Ki-67 over the time of 0, 24, and 48 hours after scratch in both groups (4T1-scramble cells and 4T1-shRNA-Gal-3 cells).
