# Supplementary material for: Galectin-3 Regulates the Expression of Tumor Glycosaminoglycans and Increases the Metastatic Potential of Breast Cancer
Source: J Oncol. 2019 Dec 17;2019:9827147. doi: 10.1155/2019/9827147 (PMC6942910; doi:10.1155/2019/9827147)
Supplement: Supplementary Materials — Supplementary Figure 1: Galectin-3 knockdown 4T1 cells did not alter the cell cycle. (A) Cell cycle analysis by flow cytometry using propidium iodide (PI) in 4T1-scramble cells and 4T1-shRNA-Gal-3 cells. Results are shown as means ± s.d. Supplementary Figure 2: cell proliferation over time in the scratch assay. Immunostaining for Ki-67 over time in the scratch assay. The red arrows indicate nuclei positive for Ki-67 over the time of 0, 24, and 48 hours after scratch in both groups (4T1-scramble cells and 4T1-shRNA-Gal-3 cells). [file 9827147.f1.docx]

**SUPPLEMENTARY MATERIALS**


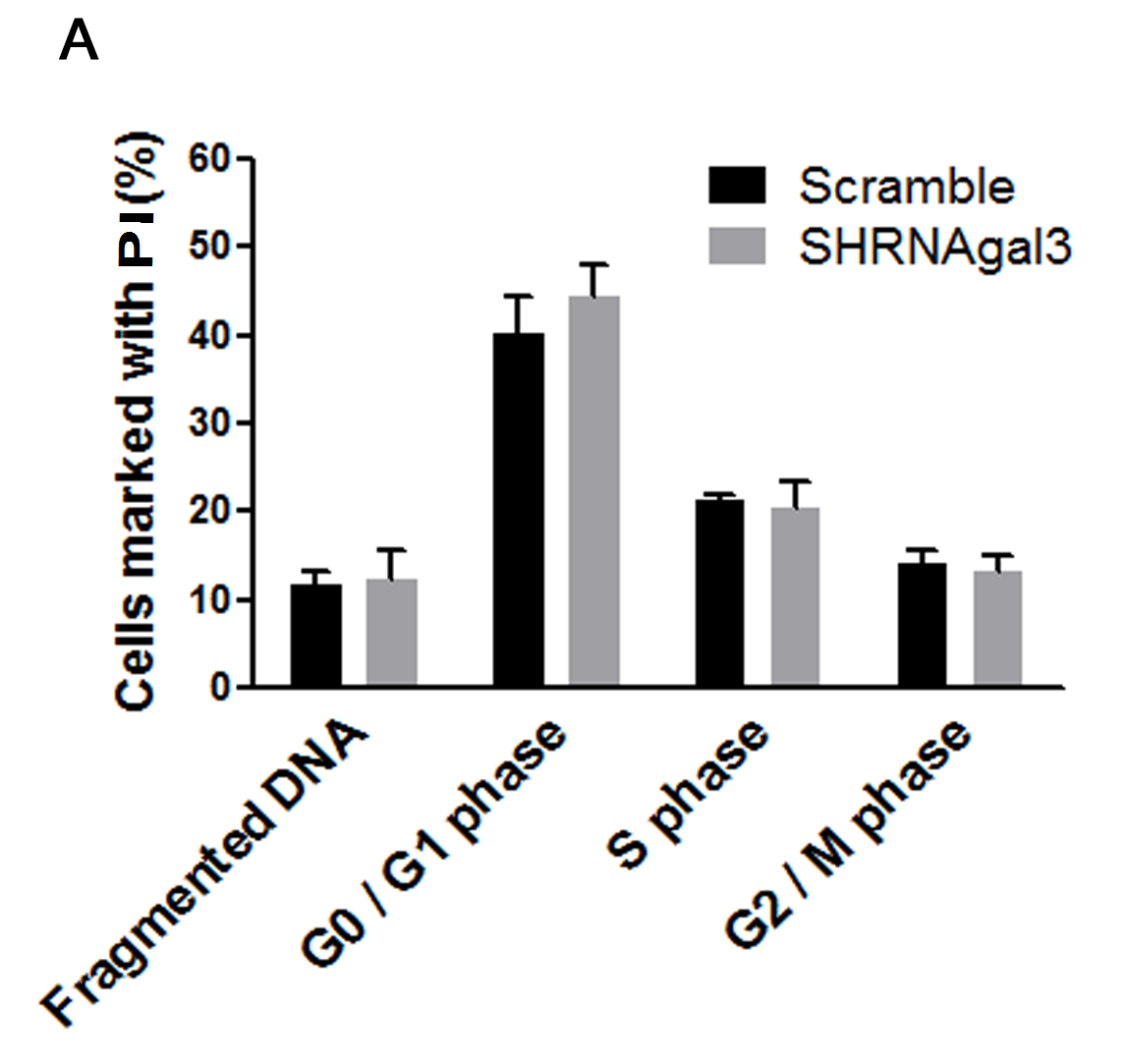


**Supplementary Figure 1: Galectin-3 knockdown 4T1 cells did not alter the cell cycle.**

**A.** Cell cycle analysis by flow cytometry using propidium iodide (PI) in scramble 4T1 cells and shRNA-Gal-3 4T1 cells. Results are shown as the means ± s.d.


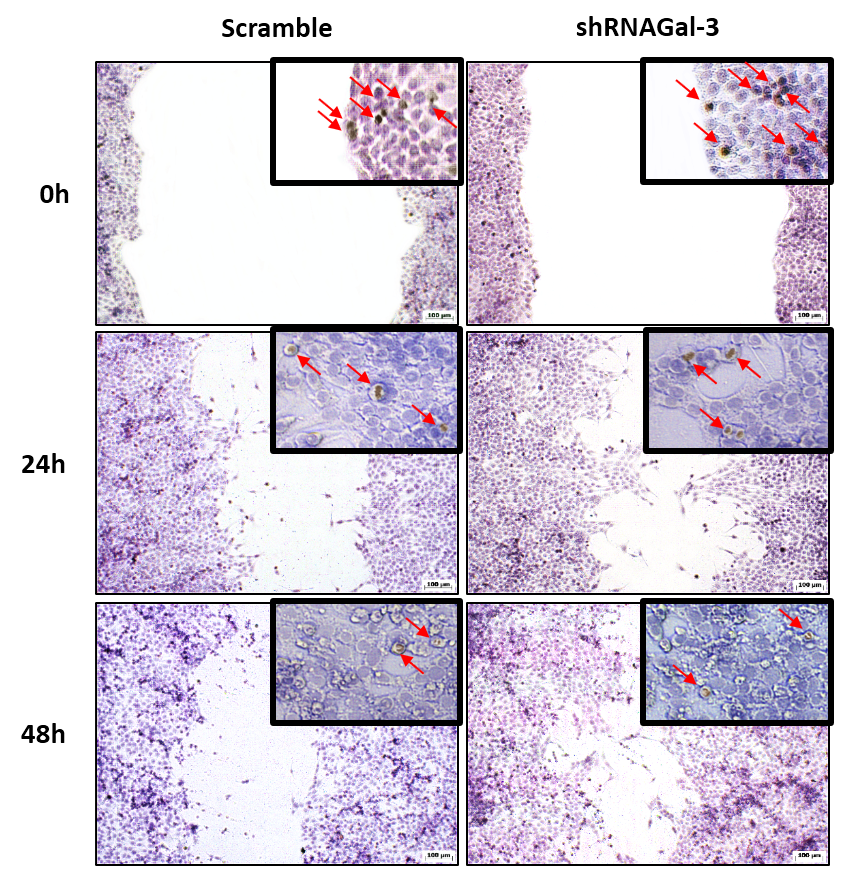


**Supplementary Figure 2: Cell proliferation over time in the scratch assay.**

Immunostaining for KI-67 over time in the scratch assay. The red arrows indicate nuclei positive for ki-67 over the times of 0, 24 and 48 hours after scratch in both groups (Scramble 4T1 cells and shRNA-Gal-3 4T1 cells).
